# Supplementary material for: Biocontrol Potential of an Endophytic Pseudomonas poae Strain against the Grapevine Trunk Disease Pathogen Neofusicoccum luteum and Its Mechanism of Action
Source: Plants (Basel). 2023 May 28;12(11):2132. doi: 10.3390/plants12112132 (PMC10255278; doi:10.3390/plants12112132)
Supplement: Supplementary file 1 [file plants-12-02132-s001.zip › plants-2348040-supplementary.pdf]

Table S1. Secondary metabolites of *P. poae* BCA strains 13, 14, 17, and JMN1 and their corresponding biosynthetic gene clusters.

Secondary Metabolite	Gene	Location	Subject cluster
**Pyoverdine**			
	PFL_4178	AAY93434.1	MbtH-like_protein
	PFL_4179	AAY93435.2	2,4-diaminobutyrate_4-transaminase
	PFL_4180	AAY93436.1	sensor_histidine_kinase
	PFL_4081	AAY93437.1	DNA-binding_response_regulator
	PFL_4082	AAY93438.1	thiol:disulfide_interchange_protein_DsbD
	PFL_4083	AAY93439.1	antioxidant,_AhpC_family
	PFL_4084	AAY93440.1	thiol:disulfide_interchange_protein_DsbG
	PFL_4089	AAY93445.1	non-ribosomal_peptide_synthetase_PvdL
	PFL_4190	AAY93446.1	RNA_polymerase_sigma-70 factor, ECF subfamily, Pvds
	PFL_4191	AAY93447.1	Pyoverdine biosyntyhesis protein
**Viscosin**			
	PFLU_2552	CAY48788.1	putative_non-ribosomal_peptide_ synthetase
	PFLU_2553	CAY48789.1	putative_non-ribosomal_peptide_ synthetase
	PFLU_2555	CAY48790.1	putative_drug-efflux_protein
	PFLU_2556	CAY48791.1	macrolide-specific_ABC-type_efflux_ carrier
	PFLU_2557	CAY48792.1	putative_LuxR-family_regulatory_protein
	PFLU_2558	CAY48793.1	methionine_gamma-lyase
	PFLU_2559	CAY48794.1	AsnC_family_regulatory_protein
**Orfamide**			
	PFL_2145	AAY91419.3	non-ribosomal_peptide_synthetase_OfaA
	PFL_2146	AAY91420.2	non-ribosomal_peptide_synthetase_OfaB
	PFL_2147	AAY91421.3	non-ribosomal_peptide_synthetase_OfaC
	PFL_2148	AAY91422.3	efflux_transporter,_RND_family,_MFP_ subunit
	PFL_2149	AAY91423.3	efflux_ABC_transporter_permease /ATP- binding_protein
	PFL_2150	AAY91424.1	transcriptional_regulator,_LuxR_family
**Putisolvin**			
	ABW17375.1	ABW17375.1	PsoA
	ABW17376.1	ABW17376.1	PsoB
	ABW17377.1	ABW17377.1	PsoC

	ABW17378.1	ABW17378.1	MacA
	ABW17379.1	ABW17379.1	MacB
	ABW17380.1	ABW17380.1	LuxR-like_protein
**Poaemide**			
	H045_07035	AGE25479.1	putative_non-ribosomal_peptide_synthetase
	H045_10940	AGE25480.1	amino_acid_adenylation_protein
	H045_10945	AGE25481.1	putative_drug-efflux_protein
	H045_07050	AGE25482.1	macrolide-specific_ABC-type_efflux_carrier
	H045_10955	AGE25483.1	putative_LuxR_family_regulatory_protein
**Anikasin**			
	A8O26_RS1 3955	WP_06411855 7.1	MacB_family_efflux_pump_subunit
	A8O26_RS1 3960	WP_06411855 8.1	macrolide_transporter_subunit_MacA
	A8O26_RS1 3965	WP_06411855 9.1	non-ribosomal_peptide_synthetase
	A8O26_RS1 3970	WP_06411856 0.1	non-ribosomal_peptide_synthetase
	A8O26_RS1 3975	WP_06411856 1.1	non-ribosomal_peptide_synthetase
	A8O26_RS1 3980	WP_06411856 2.1	non-ribosomal_peptide_synthetase
**Rhizoxin**			
	CAL69886.1	CAL69886.1	RhiI_protein
	CAL69887.1	CAL69887.1	RhiG_protein
	CAL69888.1	CAL69888.1	RhiA_protein
	CAL69889.1	CAL69889.1	RhiB_protein
	CAL69890.1	CAL69890.1	RhiC_protein
	CAL69891.1	CAL69891.1	RhiD_protein
	CAL69892.1	CAL69892.1	RhiH_protein
	CAL69893.1	CAL69893.1	RhiE_protein
	CAL69894.1	CAL69894.1	RhiF_protein
**WLIP**			
	AFJ23825.1	AFJ23825.1	WLIP_synthetase_B
	AFJ23826.1	AFJ23826.1	WLIP_synthetase_C
	AFJ23827.1	AFJ23827.1	MacA-like_protein
	AFJ23828.1	AFJ23828.1	MacB-like_ABC_transporter_protein
**Cichopeptin**			
	AHZ34239.1	AHZ34239.1	CipB
	AHZ34242.1	AHZ34242.1	CipE
	AHZ34248.1	AHZ34248.1	MacA
	AHZ34249.1	AHZ34249.1	MacB
**Tolaasin**			
	CCJ67637.1	CCJ67637.1	TaaB
	CCJ67640.1	CCJ67640.1	TaaE
	CCJ67641.1	CCJ67641.1	macrolide-specific_efflux_protein_macA

	CCJ67642.1	CCJ67642.1	macrolide_export_ATP-binding/permease_protein_MacB
**Sessilin**			
	AFH75320.1	AFH75320.1	nonribosomal_peptide_synthetase
	AFH75321.1	AFH75321.1	nonribosomal_peptide_synthetase
	AFH75323.1	AFH75323.1	macrolide-specific_efflux_protein
	AFH75324.1	AFH75324.1	macrolide_export_ATP-binding/permease_protein_MacB
**Bananamides**			
	AOA33121.1	AOA33121.1	nonribosomal_peptide_synthetase
	AOA33122.1	AOA33122.1	nonribosomal_peptide_synthetase
	AOA33123.1	AOA33123.1	nonribosomal_peptide_synthetase
	AOA33124.1	AOA33124.1	RND_family_efflux_transporter_MFP_subunit
	AOA33125.1	AOA33125.1	ABC_transporter_ATP-binding_protein
	AOA33126.1	AOA33126.1	LuxR_family_DNA-binding_response_regulator
**Entolysin**			
	PSEEN3042	CAK15812.1	macrolide_ABC_efflux_protein_MacB
	PSEEN3043	CAK15813.1	macrolide_efflux_protein_MacA
	PSEEN3044	CAK15814.1	putative_non-ribosomal_peptide_synthetase,_terminal_component
	PSEEN3045	CAK15815.1	putative_non_ribosomal_peptide_synthetase
**Xantholysin**			
	AGM14933.1	AGM14933.1	xantholysin_synthetase_B
	AGM14934.1	AGM14934.1	xantholysin_synthetase_C
	AGM14935.1	AGM14935.1	MacA-like_protein
	AGM14936.1	AGM14936.1	MacB-like_ABC_transporter_protein
**Syringafactin**			
	PSPTO_282 9	AAO56328.1	non-ribosomal_peptide_synthetase_SyfA
	PSPTO_283 0	AAO56329.1	non-ribosomal_peptide_synthetase_SyfB
	PSPTO_283 1	AAO56330.1	syringafactin_efflux_protein_SyfC
	PSPTO_283 2	AAO56331.1	syringafactin_efflux_protein_SyfD
**Arthrofactin**			
	BAC67534.2	BAC67534.2	arthrofactin_synthetase_A
	BAC67535.1	BAC67535.1	arthrofactin_synthetase_B
	BAC67536.1	BAC67536.1	arthrofactin_synthetase_C
	BAC67537.1	BAC67537.1	putative_periplasmic_protein

**APE Vf**			
	VF_084155	AAW85336.1	Dehydrogenase
	VF_084662	AAW85341.1	acyl_carrier_protein
	VF_084753	AAW85342.1	acyl_carrier_protein
	VF_085154	AAW85346.1	predicted_acyltransferase
	VF_085263	AAW85347.1	phenylalanine_and_histidine_ammonia-lyase
	VF_085346	AAW85348.1	Esterase
	VF_085751	AAW85352.1	3-oxoacyl-(acyl_carrier_protein)_synthase
	VF_086061	AAW85355.1	3-oxoacyl-(acyl_carrier_protein)_synthase
**APE Ec**			
	c1186	AAN79648.1	Putative_beta-ketoacyl-ACP_synthase
	c1187	AAN79649.1	3-oxoacyl-[acyl-carrier_protein]_reductase
	c1189	AAN79651.1	Putative_3-oxoacyl-[ACP]_synthase
	c1193	AAN79655.1	Hypothetical_protein
	c1194	AAN79656.1	Putative_enzyme
	c1199	AAN79661.1	Putative_acyl_carrier_protein
	c1200	AAN79662.1	Putative_acyl_carrier_protein
